# Does Workforce Participation After Retirement Age Affect the Use of Healthcare Services?

**DOI:** 10.3390/healthcare14121655

**Published:** 2026-06-11

**Authors:** Liqing Li, Jiashan Teng, Haifeng Ding

**Affiliations:** 1 College of Public Administration and Law, Hunan Agricultural University, Changsha 410128, China; liliqing1136@163.com; 2 School of Humanities and Management, Hunan University of Chinese Medicine, Changsha 410128, China

**Keywords:** workforce participation after retirement age, healthcare utilization, older adults, delayed retirement, China, China Health and Retirement Longitudinal Study (CHARLS) data

## Abstract

**Highlights:**

**What are the main findings?**

- Using CHARLS 2018 data, this study finds that workforce participation after retirement age is significantly associated with lower healthcare utilization among older adults in China.
- This lower utilization is primarily driven by tighter time constraints for seeking care and differences in income levels, explicitly rather than reflecting an actual improvement in the underlying health status of the working population.
- This negative association exhibits heterogeneity, varying significantly across sub-populations based on gender, self-rated health status, and educational attainment.

**What are the implications of the main findings?**

- The findings highlight a potential unintended consequence of delayed retirement policies: older adults may face unmet medical needs due to occupational time conflicts.
- Policymakers should adopt flexible and differentiated measures to better reconcile continued labor participation with accessible healthcare for older adults.

**Abstract:**

**Background:** Population ageing and the gradual implementation of delayed retirement policies have drawn increasing attention to the lives of older adults after retirement age. Although previous studies have examined the relationship between retirement and health outcomes, limited evidence is available on whether continued workforce participation after retirement age affects healthcare utilization. **Methods:** Using nationally representative data from the 2018 China Health and Retirement Longitudinal Study (CHARLS), we employ negative binomial regression as the baseline model and use an instrumental variable two-stage least squares (IV-2SLS) to address endogeneity. We further conduct heterogeneity and mechanism analyses. **Results:** The findings reveal that workforce participation after retirement age significantly reduces healthcare utilization: post-retirement workers have 42.1% fewer outpatient visits and 49.2% fewer inpatient admissions than their fully retired counterparts. Mechanism analyses indicate that the negative effect operates primarily through tighter time constraints that crowd out care-seeking time and income fluctuations that alter health investment behaviors. Heterogeneity analyses further show that the reduction in outpatient utilization is more pronounced among males and highly educated individuals, whereas the reduction in inpatient utilization is stronger for females and those with good self-rated health. **Conclusions:** Workforce participation after retirement age may hinder healthcare utilization among older adults. These findings reveal an unintended consequence of delayed retirement policies and call for flexible, targeted arrangements to balance labor participation and healthcare access for older workers.

## 1. Introduction

In contemporary society, the trend of population aging is increasingly evident, with the living conditions and health issues of older adults commanding significant attention globally. Retirement is typically regarded as a pivotal life stage, marking the conclusion of a primary working career [1]. However, China’s retirement system and labor market dynamics for older adults differ markedly from those in Western countries. China has maintained one of the lowest statutory retirement ages in the world: 60 for men, 55 for female white-collar workers, and 50 for female blue-collar workers [2]. This contrasts sharply with most Western nations, such as Germany, Sweden, and the US, where the retirement age is typically standardized between 65 and 67 for both genders [3]. Consequently, “working after retirement age” in China frequently involves individuals in their 50s and early 60s. To address accelerating population aging and shifting labor market dynamics, continuous policy adjustments have been prompted in China. Notably, on 13 September 2024, a decision was adopted to gradually delay the statutory retirement age over a 15-year period, commencing 1 January 2025. Under this decision, the statutory retirement age will progressively increase from 60 to 63 years for men, and from 50 and 55 years to 55 and 58 years for women [4].

Driven by economic pressures, or a desire for self-actualization, individuals who have reached these statutory age thresholds often choose to remain in or re-enter the labor market, a phenomenon defined as workforce participation after retirement age [2,5]. Compared to Western countries, the labor force participation rate among older adults in China is relatively high, sharing more similarities with other East Asian nations like Japan and South Korea, where post-retirement employment is heavily relied upon to supplement income or maintain social engagement [6]. According to the Seventh National Population Census, individuals aged 60 and above constituted 8.8% of the total employed population in China, representing a 5.4 percentage point increase compared to the previous census [7]. It must be clarified that within the Chinese context, a significant portion of the population—particularly rural residents and agricultural workers never formally participated in the urban formal labor market and thus do not experience a traditional “retirement transition.” As this phenomenon of continued employment becomes more prevalent, it is recognized that older adults contribute to the economy by addressing labor shortages and sustaining tax revenue [8]. However, during this process, their lifestyles, health status, and utilization of healthcare services inevitably undergo corresponding variations.

Healthcare utilization is a crucial indicator for assessing health status and the efficacy of medical security systems [9]. It has been demonstrated in numerous studies that conventional retirement often correlates with a sudden increase in individual healthcare utilization, placing mounting pressure on medical insurance funds [10]. Consequently, analyzing how workforce participation after retirement age relates to healthcare utilization holds significant practical implications for the rational allocation of medical resources. Historically, in research concerning retirement, focus has primarily been placed on intermediate mechanisms affecting healthcare use, such as changes in health status [11], time costs [12], income levels [13], and insurance reimbursement rates [14].

Regarding workforce participation after retirement age specifically, existing literature has predominantly focused on its broader psychosocial and familial outcomes. Rather than directly addressing healthcare utilization, previous studies have thoroughly documented how such participation is associated with subjective well-being and mental health [15], physical frailty [16], and perceived caregiving needs [17]. Furthermore, extensive research has been conducted on its intergenerational effects, noting that time spent on working often crowds out grandparental caregiving time, which is subsequently associated with a reduction in offspring fertility [18]. Motivation for this continued labor engagement has also been categorized into financial necessity for lower-income groups and self-actualization for those with higher socioeconomic status [19].

Despite this comprehensive exploration of socioeconomic and psychological outcomes, a consensus on the specific relationship between workforce participation after retirement age and healthcare utilization is lacking. To address this gap, data from the 2018 China Health and Retirement Longitudinal Study (CHARLS) were utilized in this study to systematically analyze the correlation between workforce participation after retirement age and healthcare utilization, along with the underlying mechanisms. The objective is to provide scientific evidence for government policy formulation, the improvement of older adult care services, and the enhancement of self-directed health management within the unique demographic and institutional context of China.

## 2. Literature Review and Research Hypotheses

Conventional retirement is often associated with a sudden increase in healthcare use [20]. However, compared to those who fully withdraw from the labor market, older adults involved in workforce participation after retirement age maintain occupational activity and social identity. Such engagement may mitigate the social isolation often triggered by retirement, preventing the excessive focus on personal health issues that sometimes leads to over-utilization of healthcare services [21]. Consequently, by sustaining physical and mental activity, older adults may experience a reduction in overall healthcare demand. Based on this, the following hypothesis is proposed:

**Hypothesis** **1.** *Workforce participation after retirement age reduces healthcare utilization among older adults.*

Within the framework of time allocation theory, focus is placed on how individuals distribute finite time across labor, domestic tasks, and leisure [22]. For older adults engaged in workforce participation after retirement age, the expansion of working hours necessitates a structural compression of non-work activities [23]. This compression primarily impacts flexible activities with high time elasticity, such as healthcare visits, which often involve unpredictable waiting and travel times [24]. When time becomes scarce, the opportunity cost of seeking medical care rises, potentially compelling individuals to postpone non-urgent appointments or forgo preventive check-ups.

Furthermore, workforce participation after retirement age may introduce “passive health behaviors”, such as physical exertion during commuting or work tasks, which may mask the perception of health risks [25]. Crucially, the reduction in healthcare utilization via this mechanism does not stem from improved health status but rather from time constraints [26]. This leads to a state of unmet medical needs, where healthcare is under-utilized despite existing health risks. Such a reduction may diminish long-term welfare by masking underlying issues. Therefore, the following hypothesis is proposed:

**Hypothesis** **2.** *Workforce participation after retirement age reduces healthcare utilization among older adults by increasing working hours, reducing leisure time, and increasing passive health-related activities.*

In contrast to the passive reduction described above, the Health Belief Model suggests that workforce participation after retirement age can actively improve health through structured routines [27]. Regular work schedules often encourage more disciplined lifestyles, including increased physical activity, improved dietary habits, and a reduction in sedentary behaviors. These proactive health adjustments directly lower the risk of chronic and acute diseases, thereby diminishing the actual need for healthcare services at the source [21]. Based on this, the following hypothesis is proposed:

**Hypothesis** **3.** *Workforce participation after retirement age reduces healthcare utilization among older adults by promoting healthier and more regular lifestyles.*

According to health human capital theory, health is an investable stock determined by the allocation of time and financial resources [28]. Workforce participation after retirement age serves as a critical pillar for accumulating this capital by providing sustained labor income. Increased income relaxes budgetary constraints, enabling older adults to invest in superior nutrition, better living environments, and high-quality preventive care [29]. Through the accumulation of health capital, the incidence of disease is lowered, leading to a rational decrease in reactive healthcare demand [30]. Based on this, the following hypothesis is proposed:

**Hypothesis** **4.** *Workforce participation after retirement age reduces healthcare utilization among older adults by raising income levels and increasing health investment.*

## 3. Research Design

### 3.1. Data Sources

The data used in this study are drawn from the China Health and Retirement Longitudinal Study (CHARLS), a nationwide household survey organized and conducted by the National School of Development at Peking University. The survey targets Chinese households with members aged 45 and above. The baseline survey was launched in 2011, with biennial follow-ups covering 28 provinces, 150 county-level units, and 450 community (village) units nationwide. CHARLS collects comprehensive household- and individual-level data [31]. The questionnaire covers retirement and employment of older adults, healthcare utilization, health status and functional capacity, healthcare provision, and pension insurance. These data are rich and comprehensive, well-suited to the objectives of this study. This study uses the 2018 wave of CHARLS data. Because this study focuses on the impact of workforce participation after retirement age on healthcare utilization among older adults, the sample is restricted to individuals who have reached the statutory retirement age (60 for men, 50 for female blue-collar workers, and 55 for female white-collar workers). After excluding observations with missing values and outliers, the final analytical sample consists of 2400 valid observations.

### 3.2. Variable Selection and Sample Description

#### 3.2.1. Dependent Variable

Medical services encompass both outpatient and inpatient care. In the CHARLS questionnaire, these are captured through the following two questions: (1) “How many times did you attend an outpatient clinic in the past month?” and (2) “How many times did you receive inpatient treatment in the past year?” Given that the two measures differ in their recall periods and reflect different aspects of healthcare utilisation, this study uses the number of outpatient visits in the past month and the number of inpatient admissions in the past year as two separate dependent variables, and estimates separate models for each [32].

#### 3.2.2. Explanatory Variables

The core explanatory variable in this study is workforce participation after the statutory retirement age. Following the sample restriction described in Section 3.1, the analytical sample is limited to individuals who have reached the statutory retirement age in China (60 for men, 50 for female blue-collar workers, and 55 for female white-collar workers). Within this sample, an individual is defined as engaging in post-retirement work if they satisfy any of the following conditions: (i) engaged in agricultural production activities for 10 or more days in the past year; (ii) engaged in at least one hour of paid work in the past week; or (iii) currently on temporary leave, training, or study while employed [7].

#### 3.2.3. Control Variables

Drawing upon relevant literature, the control variables in this study primarily encompass three aspects: individual propensity characteristics, need variables, and enabling variables among middle-aged and elderly individuals [30,31,33]. Among these, individual propensity characteristics include age, gender, place of residence, educational attainment, and marital status. Given that the physical health status of middle-aged and elderly individuals directly influences healthcare service utilisation, variables such as chronic diseases and self-rated health were selected for the need variable category. Self-rated health status was collected via questionnaire, with the original options categorised into five levels: “very good”, “good”, “fair”, “poor”, and “very poor”. Drawing on existing research, this study combines the “poor” and “very poor” categories into “poor self-rated health status,” while the remaining categories are uniformly defined as “good self-rated health status.” Enabling variables capture the financial and institutional resources that facilitate older adults’ access to healthcare services. Two variables are included: health insurance and personal income. Health insurance is measured as a categorical variable with four categories: no medical insurance, employee medical insurance, resident medical insurance, and other medical insurance. Personal income is measured as the individual’s annual net income from all sources and is log-transformed to mitigate the influence of extreme values.

#### 3.2.4. Mediating Variable

First, regarding the time allocation mechanism, three variables were constructed to capture the restructuring of daily routines. Working hours were measured using the questionnaire item “average number of working days per week.” Leisure time was operationalized based on the breadth and frequency of social engagement using data from the 2018 CHARLS. The breadth of social activities was measured via 11 dichotomous items (0 = did not participate, 1 = participated) derived from the question, “In the past month, did you engage in the following social activities?” The frequency of these activities was assessed using a three-point scale (1 = not often, 2 = almost weekly, 3 = almost daily). The final leisure time indicator was computed by summing the products of the participation status and the corresponding frequency score for each of the 11 activities. Additionally, passive health activity was represented by the self-reported frequency of light physical work, which specifically includes activities such as walking from one place to another during work.

Second, to assess the health behavior mechanism, lifestyle habits reflecting proactive health management were selected as indicators. Smoking behavior was determined by a dichotomous variable based on the question, “Do you currently smoke?” Drinking frequency was utilized to evaluate alcohol consumption over the past year. Responses to the question “How frequently did you consume alcohol in the past year?” were assigned ordinal values: 1 (no alcohol consumption), 2 (less than once per month), and 3 (more than once per month).

Third, within the context of the health human capital mechanism, variables reflecting financial resources and corresponding health investments were established. Personal income was measured by calculating the natural logarithm of the total wage income and transfer payments received over the preceding year. Finally, health investment capacity was represented by healthcare expenditure, which was calculated using the natural logarithm of total household spending on healthcare-related items during the same one-year period.

### 3.3. Model Specification

The dependent variables—outpatient visits and inpatient admissions—are non-negative count variables exhibiting overdispersion, violating the assumptions of both OLS and Poisson regression. Accordingly, we estimate the following Negative Binomial regression model [34]. The regression model is constructed as follows:

$$ln[E(Y_{i}|X_{i})]=\beta_{0}+\beta_{1}{Retired\text{\_}Status}_{i}+r^{\prime }{Control}_{i}+\delta_{r}+\varepsilon_{i}$$

where *Y_i_* is the healthcare utilisation outcome of individual *i*; *Retired_Status_i_* is a binary indicator for post-retirement work; *Control_i_* denotes the control variables defined in Section 3.2.3; *δ_r_* represents provincial fixed effects; and ε_i_ is the error term. Robust standard errors are used throughout, and exp(*β*_1_) is interpreted as the Incidence Rate Ratio (IRR).

## 4. Empirical Results Analysis

### 4.1. Descriptive Statistical Analysis

Descriptive statistics for key variables are presented in Table 1. The mean value for workforce participation after retirement age among middle-aged and older adults was 0.247, which is indicative of the fact that only a minority engaged in such behavior. The frequency of healthcare service utilization in the preceding year ranged from 0 to 30 visits, with a mean of 0.757 and standard deviation of 1.786, which is suggestive of generally low utilization rates and considerable variation among this demographic. The mean age of the study sample was 64.07 years, with all other control variables falling within normal ranges, confirming the appropriateness of variable selection.

### 4.2. Analysis of Benchmark Regression Results

Table 2 presents the negative binomial regression results on the association of workforce participation after retirement age with healthcare service utilization. Models 1 and 2 provide estimates for outpatient visits, while Models 3 and 4 pertain to inpatient admissions. Within each pair, Models 1 and 3 include only the core explanatory variable, whereas Models 2 and 4 sequentially incorporate the full set of control variables.

The coefficient for workforce participation after retirement age is negative and statistically significant at the 1% level across all four specifications, suggesting that workforce participation after retirement age is correlated with substantially lower healthcare utilization. In the fully specified models, the coefficient is −0.547 (Model 2) for outpatient visits and −0.678 (Model 4) for inpatient admissions. Translating these estimates into Incidence Rate Ratios (IRRs), older adults who engage in workforce participation after retirement age exhibit approximately 42.1% fewer outpatient visits (exp(−0.547) = 0.579) and 49.2% fewer inpatient admissions (exp(−0.678) = 0.508) than their fully retired counterparts. The larger magnitude of the inpatient coefficient is suggestive of a stronger link between workforce participation after retirement age and lower frequencies of severe or acute care episodes compared to routine outpatient care.

The estimated coefficients of the control variables are largely consistent with theoretical expectations. Self-rated health is strongly and negatively associated with both outpatient (*p* < 0.01) and inpatient utilization (*p* < 0.01), confirming that healthier individuals tend to consume fewer medical services. Chronic disease status is significantly and positively correlated with inpatient admissions (*p* < 0.01) but shows no significant relationship with outpatient visits. Educational attainment is negatively associated with inpatient utilization ( *p*< 0.05), possibly reflecting better health literacy among more educated individuals, while rural residence shows a positive link to inpatient admissions (*p* < 0.10). Health insurance shows a weakly positive association with inpatient utilization, with Employee Medical Insurance being marginally significant ( *p* < 0.10).

Overall, the benchmark results provide robust evidence that workforce participation after retirement age is significantly associated with lower outpatient and inpatient healthcare service utilization among older adults in China, with the relationship being more pronounced for inpatient services.

### 4.3. Endogeneity Analysis

To address potential endogeneity arising from omitted variables and the health selection effect (i.e., healthier individuals being more likely to work), an Instrumental Variable (IV) approach using Two-Stage Least Squares (2SLS) was employed. Following the previous literature, the community-level workforce participation rate after retirement age was selected as the instrument [35].

As shown in Table 3, in the first-stage regression, the coefficient of the instrumental variable is significantly positive (β = 1.480, *p* < 0.01), indicating a strong peer effect: a higher employment rate among older adults in the community significantly increases the individual’s probability of working. The first-stage F-statistic is 356.860, well above the rule-of-thumb threshold of 10, confirming the absence of a weak instrument problem.

In the second-stage regression, using the exogenous variation in individual workforce participation fitted by the instrument, the results show a significant negative impact on both outpatient (β = −0.567, *p* < 0.1) and inpatient visits (β = −0.304, *p* < 0.05). This finding is highly consistent with the baseline regression, suggesting that even after mitigating the endogeneity bias, workforce participation after retirement age genuinely and significantly reduces healthcare utilization among older adults.

### 4.4. Robustness Test

To verify the reliability of the baseline findings, three sets of robustness checks were conducted, with results reported in Table 4. First, in Models 1 and 2, the baseline negative binomial regression is replaced with a Poisson regression model. The coefficient on workforce participation after retirement age remains negative and statistically significant at the 1% level for both outpatient visits (β = −0.584, *p* < 0.01) and inpatient visits (β = −0.716, *p* < 0.01), consistent with the baseline estimates. Second, in Models 3 and 4, healthcare visit counts are replaced with out-of-pocket expenditure as alternative outcome variables. Workforce participation after retirement age remains negatively associated with outpatient costs (β = −0.248, *p* < 0.05) and inpatient costs (β = −0.464, *p* < 0.01), suggesting that workforce participation after retirement age is inversely correlated with not only the frequency but also the financial burden of healthcare utilization. Third, in Models 5 and 6, winsorization is applied at the 1st and 99th percentiles to address potential outlier bias. The estimated associations for outpatient visits (β = −0.547, *p* < 0.01) and inpatient visits (β = −0.678, *p* < 0.01) remain virtually unchanged relative to the baseline.

Across all three robustness checks, the negative and statistically significant relationship between workforce participation after retirement age and healthcare utilization is consistently maintained, confirming the reliability of the baseline findings.

## 5. Further Analysis

### 5.1. Heterogeneity Analysis

To examine whether the association between workforce participation after retirement age and healthcare utilization varies systematically across population subgroups, the sample is stratified by gender, self-rated health status, and educational background. Results are reported in Table 5.

#### 5.1.1. Gender Heterogeneity

The full sample is divided into male (n = 1209) and female (n = 1191) subgroups. Among males, workforce participation after retirement age is negatively and significantly associated with both outpatient visits (β = −0.873, *p* < 0.01) and inpatient admissions (β = −0.585, *p* < 0.01). Among females, a significant negative relationship is observed for inpatient admissions (β = −0.929, *p* < 0.01), whereas the coefficient for outpatient visits is negative but falls short of statistical significance (β = −0.109, *p* > 0.10). These findings indicate that the negative correlation between workforce participation after retirement age and outpatient care-seeking differs notably by gender.

#### 5.1.2. Heterogeneity by Self-Rated Health Status

The sample is stratified by self-rated health into poor health (n = 402) and good health (n = 1998) subgroups. Among individuals reporting poor health, workforce participation after retirement age is negatively associated with outpatient visits (β = −0.512, *p* < 0.10), while the coefficient for inpatient admissions is negative and statistically insignificant (β = −0.233, *p* > 0.10). Among those in good health, workforce participation after retirement age is negatively associated with both outpatient visits (β = −0.544, *p* < 0.05) and inpatient admissions (β = −0.884, *p* < 0.01). The estimated associations are statistically stronger and more consistent across both utilization measures in the good health subgroup than in the poor health subgroup.

#### 5.1.3. Heterogeneity in Educational Background

The sample is divided by educational attainment into two subgroups: primary school and below (n = 819) and secondary education and above (n = 1581). In the lower education subgroup, workforce participation after retirement age is negatively associated with outpatient visits (β = −0.515, *p* < 0.10) and inpatient admissions (β = −0.807, *p* < 0.01). In the higher education subgroup, statistically significant negative relationships are observed for both outpatient visits (β = −0.649, *p* < 0.01) and inpatient admissions (β = −0.607, *p* < 0.01). The magnitude of the association for inpatient admissions is larger among the lower education group, while the correlation for outpatient visits is more pronounced among those with secondary education and above.

### 5.2. Mechanism Analysis

#### 5.2.1. Time Constraint Mechanism

To systematically validate the time constraint mechanism, working hours, leisure time, and passive health activities are analyzed as mediating variables. As shown in Models 1 and 2 of Table 6, workforce participation after retirement age is associated with higher working hours (β = 1.999, *p* < 0.01) and lower leisure time (β = 0.443, *p* < 0.05). Furthermore, the results in Model 3 reveal that workforce participation after retirement age is significantly and positively associated with passive health activities (β = 0.451, *p* < 0.01).

#### 5.2.2. Health Behaviour Mechanism

To systematically examine the health behavior mechanism, smoking behavior and drinking frequency are selected as mediating variables. As shown in Models 4 and 5 of Table 6, workforce participation after retirement age is negatively associated with both smoking behavior (β = −0.187, *p* > 0.10) and drinking frequency (β = −0.052, *p* > 0.10). Although the direction of these coefficients is negative, neither association is statistically significant at conventional levels in this sample.

#### 5.2.3. Income Variation Mechanism

To systematically validate the income variation mechanism, personal income and healthcare program investment expenditure are analyzed as mediating variables. The regression results in Models 6 and 7 of Table 6 demonstrate that workforce participation after retirement age exhibits a positive and highly significant correlation with personal income (β = 0.168, *p* < 0.01). Additionally, workforce participation after retirement age is associated with higher healthcare expenditure, a relationship that is statistically significant at the 10% level (β = 0.364, *p* < 0.10).

## 6. Discussion

### 6.1. Summary

This study used data from the 2018 China Health and Retirement Longitudinal Study (CHARLS) to analyze the impact of workforce participation after retirement age on the use of healthcare services among older adults. By employing baseline regression, heterogeneity analysis, and mechanism tests, this research aims to identify how extended labor participation affects health-seeking behaviors. The benchmark results robustly indicate that workforce participation after retirement age significantly reduces both outpatient and inpatient healthcare service utilization among older adults in China, with the negative effect being particularly pronounced for inpatient services.

In terms of control variables, self-rated health and the presence of chronic diseases are critical correlates of healthcare utilization. As shown in the results, older adults with chronic conditions exhibit a significantly higher reliance on medical services, while those with better self-rated physical health report drastically lower utilization rates [36]. Furthermore, demographic factors such as place of residence and educational level also play pivotal roles. Urban residents generally utilize more healthcare services than their rural counterparts, largely due to superior medical infrastructure and higher accessibility in cities [37]. However, when evaluating these baseline results, an essential underlying factor must be highlighted good physical health is inherently a primary prerequisite and motivation for individuals to continue working after reaching retirement age. Because these older adults are relatively healthier and possess better physical functions, their intrinsic demand for medical treatment is naturally lower [38].

In the heterogeneity analysis, it is demonstrated that the association between workforce participation after retirement age and the use of healthcare services varies significantly across demographic groups. Specifically, the negative correlation with healthcare utilization is most pronounced among women, individuals with good self-rated health, and those with higher educational attainment. For older women, traditional societal roles often require them to balance workforce participation after retirement age with intergenerational caregiving and domestic duties [18]. This double burden severely compresses their available time, forcing them to postpone or forgo medical visits unless facing severe illness [39]. Furthermore, Our findings highlight that for older adults with higher educational attainment, post-retirement employment significantly reduces the likelihood of seeking medical services. This trend suggests that for the highly educated, the professional demands and the high intrinsic value of their work outweigh the perceived urgency of healthcare visits. In this context, the conflict between “work time” and “care-seeking time” becomes the primary driver of reduced healthcare utilization, rather than financial constraints or a lack of medical insurance [40].

The mechanism analysis further explores the pathways through which workforce participation after retirement age correlates with the use of healthcare services, primarily pointing to reduced time available for seeking care, and income fluctuations. Unlike fully retired older adults who enjoy abundant leisure time, working older adults face strict time constraints. The high opportunity cost of taking time off for outpatient visits or inpatient care is negatively correlated with their immediate medical needs [22]. It is noteworthy that specific lifestyle habits, namely smoking and alcohol consumption, do not exhibit significant mediating associations. This insignificance can largely be attributed to the deeply ingrained nature of these behaviors. For most older adults, smoking and drinking are long-term habits or addictions established decades earlier in their youth or middle age. Consequently, these behaviors are highly path-dependent and resistant to short-term variations associated merely with workforce participation after retirement age [41]. Finally, the additional income derived from post-retirement work plays a crucial role in improving health outcomes [42]. Older adults utilize these continuous earnings to invest in their health, thereby maintaining a higher level of physical well-being. This enhanced health status, driven by financial security, naturally diminishes their overall vulnerability to disease, which in turn leads to a decreased frequency of outpatient visits and hospital admissions.

In developed economies confronting severe demographic aging, such as Japan and several European nations, workforce participation after retirement age has become a mainstream strategy to mitigate the “silver tsunami” [43]. International research indicates that while workforce participation after retirement age can preserve cognitive function and foster social integration, it demands highly flexible workplace policies to accommodate the inevitable medical needs of aging employees [44]. In Western welfare states, comprehensive social safety nets, generous paid medical leave, and flexible working hours facilitate a relative balance between workforce participation after retirement age and healthcare access [45]. In contrast, older adults in China, particularly those re-employed in the informal sector, face more rigid trade-offs between working and seeking medical treatment. Therefore, as global life expectancy continues to rise, the Chinese experience underscores a universal implication: policymakers and employers must design age-friendly work environments and adopt differentiated support measures to ensure that older adults are not implicitly penalized for seeking necessary healthcare services while contributing to the economy.

### 6.2. Policy Implications

The findings of this analysis reveal the complex effects of the “workforce participation after retirement age” phenomenon on healthcare service utilisation among older adults. In light of this, to better address this phenomenon, provide more appropriate safeguards for middle-aged and elderly individuals, and lay the groundwork for the smooth implementation of future delayed retirement policies, the following policy implications are proposed.

First, within the context of delayed retirement policies, a more comprehensive healthcare security system should be established, fully accounting for the unique circumstances of retired yet still working older adults. Increased investment in healthcare resources is required, particularly in regions and industries with higher concentrations of this demographic. Targeted expansion of medical facilities and service provision should enhance accessibility and convenience, thereby mitigating the negative impact of continued employment on healthcare utilisation among this group.

Secondly, in formulating and implementing policies to delay retirement, greater consideration should be given to the differences among older adults based on gender, self-rated health, and educational background, thereby developing more targeted policy measures. To ensure equitable healthcare access, healthcare strategies should focus on men, the highly educated, and those in good health, as these groups are more likely to reduce their medical service use while working after retirement. Enhancing health literacy and optimizing healthcare resource allocation for these individuals can help bridge the gap between labor participation and health maintenance.

Thirdly, implementing flexible retirement and employment systems can mitigate time constraint effects. Policies should focus on enhancing autonomy and flexibility in work arrangements for older workers. It is recommended that employers, particularly those in community services, public welfare positions, and small-to-medium enterprises, widely establish “flexible working hours” or part-time roles, permitting older individuals to adjust their work pace according to their health status. Concurrently, human resources departments could explore establishing a “flexible employment certification and management system for older workers”, incorporating regular health check-ups and outpatient follow-up appointments into the scope of “health-related working hours”. This would institutionally safeguard their time for medical care, preventing rigid work commitments from crowding out essential healthcare needs.

Fourthly, guide income conversion and incentivise health investment behaviour. The “workforce participation after retirement age” phenomenon demonstrates a positive pathway for converting economic capital into health human capital by stimulating healthcare expenditure through increased income. Policies should design incentive-compatible mechanisms to assist this demographic in allocating additional income towards health maintenance. For instance, collaboration with financial institutions could establish supplementary “personal health accounts” offering tax relief or modest subsidies for expenditures on medical examinations and preventive healthcare. Concurrently, commercial insurers should be encouraged to develop “health management rebate insurance” tailored to younger seniors, granting premium discounts to policyholders maintaining sound health practices or undergoing regular health screenings. This fosters a virtuous cycle: employment→increased income→health investment→health maintenance.

### 6.3. Strengths

This study offers several methodological and analytical strengths. First, it systematically examined the mediating roles of time constraints, health behaviors, and income fluctuations in the relationship between workforce participation after retirement age and healthcare utilization. This not only complements previous research that focused primarily on health outcomes but also clarifies the behavioral and economic pathways through which post-retirement work affects healthcare access. Second, the study employed an instrumental variable two-stage least squares (IV-2SLS) approach to address the endogeneity bias arising from health selection, providing more rigorous causal evidence compared to conventional observational studies. Third, this study’s analysis of heterogeneity across gender, self-rated health status, and educational attainment provides a nuanced understanding of the differential impacts of post-retirement work, offering targeted insights for policy design.

### 6.4. Limitation

The shortcomings of this study mainly include the following aspects: First, although we employed the instrumental variable two-stage least squares (IV-2SLS) approach to address potential endogeneity, the community-level instrumental variable may not fully satisfy the strict exogeneity assumption, and unobserved individual health heterogeneities may still bias the estimates. Second, the measurement of key variables has certain limitations: the core explanatory variable does not distinguish between different types, intensities and motivations of post-retirement work, while the dependent variables only measure the frequency of healthcare visits without considering service quality and type. Third, the mechanism analysis is not comprehensive, as we only tested smoking and drinking behaviors as health behavior indicators and did not explore other potential pathways such as work-related stress and social support changes.

## 7. Conclusions

Using the nationally representative micro data from the 2018 China Health and Retirement Longitudinal Study (CHARLS), this study empirically examines the impact of workforce participation after retirement age on healthcare utilization among older adults in China and further explores the underlying mechanisms and heterogeneous effects via negative binomial regression and the instrumental variable two-stage least squares (IV-2SLS) approach. The results indicate that continued workforce participation after retirement age is significantly and negatively associated with both outpatient visits and inpatient admissions among older adults. Specifically, post-retirement workers exhibit approximately 42.1% fewer outpatient visits and 49.2% fewer inpatient admissions than their fully retired peers, and these findings remain robust after addressing endogeneity and conducting multiple sensitivity tests. Heterogeneity analysis reveals that the negative association between post-retirement workforce participation and healthcare utilization varies substantially across subgroups: the adverse effect on outpatient care is more pronounced among males and individuals with higher educational attainment, while the reduction in inpatient utilization is stronger for females and those with good self-rated health status. Mechanism analysis demonstrates that the negative relationship operates primarily through two key pathways: tighter time constraints that limit care-seeking opportunities and income fluctuations that alter health investment behaviors. Notably, health behaviors such as smoking and alcohol consumption do not serve as significant mediating factors in this context. The main contributions of this study are three-fold: first, we provide rigorous empirical evidence on the causal link between workforce participation after retirement age and healthcare utilization, filling the research gap in the existing literature; second, we identify time constraints and income dynamics as critical mediating mechanisms, clarifying the behavioral and economic channels through which post-retirement work affects healthcare access; and third, we uncover subgroup disparities by gender, self-rated health, and educational attainment, offering nuanced insights for the formulation of differentiated delayed retirement policies and age-friendly healthcare interventions.

## Figures and Tables

**Table 1:** Variable Descriptions and Descriptive Statistics (N = 2400).

Variable	Variable Description	Mean ± SD	Frequency (%)
**Continuous Variables**			
Outpatient visits	Number of outpatient consultations in the past month	0.401 ± 1.501	
Inpatient visits	Number of inpatient admissions in the past year	0.344 ± 0.894	
Age	50–80 years	64.07 ± 7.443	
Personal income	Natural logarithm of annual net income from all sources for the individual	7.69 ± 0.855	
**Categorical Variables**			
Workforce participation after retirement age	Not working = 0		1763 (73.46)
	Working = 1		637 (26.54)
Gender	Male = 0		1209 (50.38)
	Female = 1		1191 (49.62)
Level of education	No education = 1		116 (4.83)
	Below primary = 2		227 (9.46)
	Primary = 3		476 (19.83)
	Secondary = 4		769 (32.04)
	Secondary and above = 5		812 (33.83)
Marital status	No spouse = 0		324 (13.50)
	Spouse present = 1		2076 (86.50)
Place of residence	Rural = 0		285 (11.87)
	Urban = 1		2115 (88.13)
Chronic disease	No chronic disease = 0		1205 (50.21)
	Chronic disease = 1		1195 (49.79)
Self-Rated Health Status	Poor = 0		402 (16.75)
	Good = 1		1998 (83.25)
Medical insurance	No medical insurance = 0		14 (0.58)
	Employee medical insurance = 1		1572 (65.50)
	Resident medical insurance = 2		517 (21.54)
	Other medical insurance = 3		297 (12.38)

Note: Continuous variables are presented as mean ± standard deviation. Categorical variables are presented as frequency (percentage).

**Table 2:** Baseline Regression Results.

Variable	Model 1 Outpatient Visits	Model 2 Outpatient Visits	Model 3 Inpatient Visits	Model 4 Inpatient Visits
Workforce participation after retirement age	−0.595 ***	−0.547 ***	−0.929 ***	−0.678 ***
	(0.184)	(0.162)	(0.136)	(0.134)
Age		−0.004		0.010
		(0.010)		(0.008)
Gender		−0.039		−0.143
		(0.142)		(0.112)
Level of education		0.082		−0.117 **
		(0.066)		(0.047)
Marital status		−0.128		0.077
		(0.222)		(0.145)
Place of residence		−0.255		0.828 *
		(0.407)		(0.431)
Chronic disease		0.171		0.787 ***
		(0.140)		(0.117)
Self-Rated Health Status		−0.772 ***		−1.046 ***
		(0.138)		(0.109)
Personal Income		0.082 ***		0.053 **
		(0.024)		(0.022)
Medical insurance (reference group: no medical insurance)				
Employee Medical Insurance		0.611		0.451 ***
		(0.631)		(0.252)
Resident Medical Insurance		0.672		0.302 ***
		(0.646)		(0.220)
Other medical insurance		1.020		0.202 ***
		(0.665)		(0.305)
Province	Control	Control	Control	Control
Constant term	−0.786 ***	0.935	−0.893 ***	−15.640 ***
	(0.085)	(0.676)	(0.057)	(0.981)
Sample size	2400	2400	2400	2400
Pseudo R^2^	0.004	0.036	0.015	0.150

Note: *** *p* < 0.01, ** *p* < 0.05, * *p* < 0.1; values in parentheses denote robust standard errors (same applies below).

**Table 3:** Endogeneity Test: Instrumental Variables Method.

Variable	Outpatient Visits		Inpatient Visits	
	Model 1	Model 2	Model 3	Model 4
	First-Stage Regression	Second-Stage Regression	First-Stage Regression	Second-Stage Regression
Community workforce participation rate after retirement age	1.480 *** (0.139)		1.480 *** (0.139)	
Workforce participation after retirement age		−0.567 * (0.338)		−0.304 ** (0.154)
Control Variables	Yes	Yes	Yes	Yes
Province	Yes	Yes	Yes	Yes
F	356.860	—	356.860	—
Adjusted R^2^	0.164	0.023	0.164	0.117

Note: Yes indicates the variable was included as a control (same applies below). *** *p* < 0.01, ** *p* < 0.05, * *p* < 0.1.

**Table 4:** Robustness Test Results.

Variable	Alternative Model (Poisson)		Alternative Outcome (Cost)		Winsorization (1–99%)	
	Model 1 Outpatient Visits	Model 2 Inpatient Visits	Model 3 Outpatient Cost	Model 4 Inpatient Cost	Model 5 Outpatient Visits	Model 6 Inpatient Visits
Workforce participation after retirement age	−0.584 *** (0.196)	−0.716 *** (0.136)	−0.248 ** (0.083)	−0.464 *** (0.091)	−0.547 *** (0.162)	−0.678 *** (0.134)
Control Variables	Yes	Yes	Yes	Yes	Yes	Yes
Province	Yes	Yes	Yes	Yes	Yes	Yes
Sample size	2400	2400	2093	2115	2400	2400
Pseudo R^2^	0.028	0.134			0.038	0.147
Adjusted R^2^			0.064	0.103		

*** *p* < 0.01, ** *p* < 0.05.

**Table 5:** Heterogeneity Analysis Results.

Variable	Gender		Self-Rated Health Status		Educational Background	
	Male	Female	Poor	Good	Primary School and Below	Secondary Education and Above
Outpatient visits	−0.873 ***	−0.109	−0.512 *	−0.544 **	−0.515 *	−0.649 ***
	(0.204)	(0.217)	(0.235)	(0.185)	(0.253)	(0.183)
Control variables	Yes	Yes	Yes	Yes	Yes	Yes
Province	Yes	Yes	Yes	Yes	Yes	Yes
Sample size	1209	1191	402	1998	819	1581
Inpatient visits	−0.585 ***	−0.929 ***	−0.233	−0.884 ***	−0.807 ***	−0.607 ***
	(0.161)	(0.266)	(0.232)	(0.161)	(0.201)	(0.178)
Control variables	Yes	Yes	Yes	Yes	Yes	Yes
Province	Yes	Yes	Yes	Yes	Yes	Yes
Sample size	1209	1191	402	1998	819	1581

*** *p* < 0.01, ** *p* < 0.05, * *p* < 0.1.

**Table 6:** Mechanism Analysis.

Variable	Time Constraint Mechanism			Health Behaviour Mechanism		Income Variation Mechanism	
	Model 1 Working Hours	Model 2 Leisure Time	Model 3 Passive Health Activities	Model 4 Smoking Behaviour	Model 5 Frequency of Alcohol Consumption	Model 6 Personal Income	Model 7 Healthcare Expenditure
Workforce participation after retirement age	1.999 *** (0.084)	−0.443 ** (0.146)	0.451 *** (0.112)	−0.187 (0.154)	−0.062 (0.052)	0.168 *** (0.053)	0.364 * (0.198)
Control Variables	Yes	Yes	Yes	Yes	Yes	Yes	Yes
Province	Yes	Yes	Yes	Yes	Yes	Yes	Yes
Sample size	1629	2018	2192	2399	2370	2118	2399
Adjusted R^2^	0.283	0.054	0.104	0.279	0.174	0.343	0.163

*** *p* < 0.01, ** *p* < 0.05, * *p* < 0.1.

## Data Availability

The original data are available at https://charls.pku.edu.cn/.
